# Dr. Marcus J. Williams

**Published:** 1917-05

**Authors:** 


**Dr. Marcus J. Williams,** Vermont 1878, died at his home in Syracuse March 11, aged 63.

